# Cancer incidence in Arkhangelskaja Oblast in northwestern Russia. The Arkhangelsk Cancer Registry

**DOI:** 10.1186/1471-2407-5-82

**Published:** 2005-07-19

**Authors:** Arild Vaktskjold, Jelena A Lebedintseva, Dmitrij S Korotov, Anatolij V Tkatsjov, Tatjana S Podjakova, Eiliv Lund

**Affiliations:** 1 Institutt for samfunnsmedisin, Universitetet i Tromsø, N-9037 Tromsø, Norway; 2 Oncological Hospital, Arkhangelsk, Russia; 3 Arkhangelsk Cancer Registry, Arkhangelsk, Russia; 4 Institute of Physiology, Arkhangelsk, Russia

## Abstract

**Background:**

Data concerning incidence and prevalence of cancer in the different regions of Russia have traditionally not been provided on a basis that facilitated comparison with data from countries in western parts of Europe. The oncological hospital in Arkhangelsk, in co-operation with Universitetet i Tromsø (Norway), has established a population based cancer registry for Arkhangelskaja Oblast (AO). AO is an administrative unit with 1.3 million inhabitants in northwestern Russia. The aim of this investigation was to assess the content and quality of the AO cancer registry (AKR), and to present the site-specific cancer-incidence rates in AO in the period 1993–2001.

**Methods:**

The population in this study consisted of all individuals registered as residents of AO. All new cancer cases in the period 1993 – 2001, registered the AKR, were included in the study (ICD-10: C00-C95, except for C77-78). The annual gender and age-group-specific population figures were obtained from the AO statistics office.

**Results:**

A total of 34 697 cases of primary cancers were included. The age-adjusted (world standard) incidence rate for all sites combined was 164/100 000 for women and 281/100 000 for men. The highest incidence was for cancer of the trachea, bronchus and lung (16.3% of all cases), whereof 88.6 % of the cases were among men. Among women, cancer of the breast constituted 15.9 percent of all cases. The age-adjusted incidences of the most frequent cancer sites among men were: lung (77.4/100 000); stomach (45.9); rectum (13.4); oesophagus (13.0); colon (12.2); bladder (11.6); and prostate cancer (11.1). Among women they were: breast (28.5); stomach (19.7); colon (12.2); and ovary cancer (9.0).

**Conclusion:**

Our findings confirm and strengthen the indication that the incidences of stomach, larynx, liver, pancreas, prostate, colon, bladder and melanoma cancer are quite different in male populations in Russia compared to many other European countries. Among women, most major cancer types, except stomach, appear to be relatively low in Russian populations. The AKR provides quality data for estimations and insight to the cancer incidence in a northern Russian population, and we consider the reported incidence rates to reflect the cancer situation in AO well.

## Background

Data concerning incidence and prevalence of cancer in the Soviet Union (USSR) and Russia have traditionally not been provided on a basis that facilitated comparison with data from countries in western parts of Europe [1]. This was due to differences in routines and procedures for reporting, registration and diagnostics [2], as well as to a lack of information that described the procedures. Today, the cancer registry in St Petersburg is the only regional cancer registration in Russia that has been recognised by the European Network of Cancer Registries to fulfil international standards, but efforts have been made to standardise the system within Russia [2]. In 1997, the oncological hospital (OD) in Arkhangelsk and the Universitetet i Tromsø (Norway) started the planning of a population based cancer registry for Arkhangelskaja Oblast (AO). The systematic registration started in 1999, and the objective was to register all cancer cases in AO from 1993 and onwards.

A verifiable cancer registry that covers the population of AO will not only give valuable insight into the cancer incidence in Russia, but also into the incidence in a northern population that has been relatively homogenous both ethnically and socio-economically. The aim of this investigation was to assess the content and quality of the AO cancer registry (AKR), and to present the site-specific cancer-incidence rates in AO in the period 1993–2001.

## Methods

### Context

**Figure 1:**
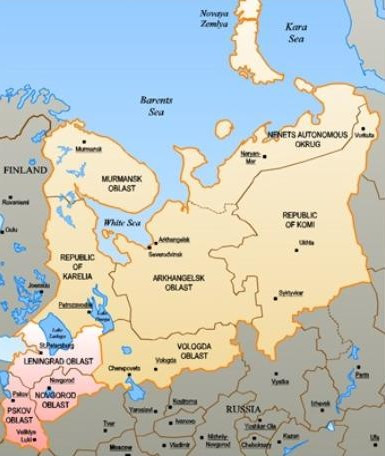
North-western Russia [17].

AO is an administrative unit with 1.3 million inhabitants. It covers 587 000 km^2 ^(larger than France) north of the 60^th ^latitude in North-West Russia (Figure 1). The unit consists of 26 boroughs, whereof 6 are cities. In addition, there is an autonomous region, Nenets, in the north. Until the late 1990s, twelve percent of the land area was for use by military units only (Nenets not included) [3]. The administrative centre is the city Arkhangelsk, which houses 28 percent of the population. The main industries are pulp and paper mills, and heat and power plants. Gas and oil industry predominate in Nenets.

The population of AO grew during the 1980s and reached a historical high of 1 576 000 inhabitants in 1991. In the years that followed, the mortality and migration increased, and the birth rate went down [4,5]. In year 2000, the largest ethnic groups were Russians (92 percent); Ukrainians (3.4); Byelorussians (1.3); Komi (0.5); and Nenets (0.5), and 74 percent of the population resided in urban areas [5]. In 1999, the life expectancy in AO was 71.1 years for women and 58.0 years for men, which was a decrease from 74.7 and 64.0, respectively, ten years earlier [5]. Life expectancy was higher in urban than in rural areas, but lower than in the neighbouring regions in North-West Russia [5,6]. The most frequent causes of death were connected to the circulatory system (54%); accidents (16%); and tumours (14%) [4].

The only oncological hospital (OD) in AO is located in the city Arkhangelsk, where 35 of the about 50 practising oncologists in 2002 worked. About one-half of the boroughs had at least one oncologist. The boroughs without an oncologist did instead have a medical worker who was responsible for cancer recording and reporting, but this person had, in general, no contact with the cancer patients. Every Russian citizen has the right to receive medical care without service fees. When a patient is referred to a district or central hospital, the local hospital might cover the travel expenses if financial resources are available. But more often than not, the patients must cover these expenses themselves. The latter is also true for persons seeking a doctor or a hospital within his/her home borough. All forms of cancer treatment are available in the city Arkhangelsk. Radiotherapy is only available there, while surgery is carried out in every hospital where there is a surgeon qualified to perform the appropriate surgery. The same is true for chemotherapy, but this treatment can only be given after the patient has been examined at the OD, and under supervision by them.

The pathology department at the OD, which is the only one in AO, has had separate laboratories for cytology and histopathology. The latter had 3 pathologists, and processed 35 000 surgical pathology specimens annually. The department had available to them a complete version of the WHO International Histological Classification of Tumours in Russian. Immunohistochemistry was established at the AO Main Hospital in 2001, in co-operation with the hospital in Tromsø. The pathology department and the main hospital co-operate closely, and frequently consult each other when faced with difficult cases.

### Study population

The population in this study consists of all individuals registered as residents of AO. All new cancer cases in the period 1993 – 2001 registered in the AKR were included in the study (International Classification of Disease, version 10 (ICD-10): C00-C95, except for C77-78 (secondary malignant neoplasm)), and the data obtained were anonymous. The estimated incidence rates of cancer were adjusted for age using the world standard (5-year age-group intervals) [7]. In instances where an individual was diagnosed with cancer more than once during a six-month period, only the first diagnosis was included in the rate estimates. When a registered diagnosis of cancer subsequently showed not to be cancer, the case was not included. The annual gender and age-group-specific population figures for AO were obtained from the federal statistics office of AO. In the calculations of cancer incidence for year 2001, population figures for year 2000 were used for the age groups older than 69 years.

### Cancer reporting and the Arkhangelsk Cancer Registry

The cancer reporting and registration is founded in legislation. The oncologist/doctor who determines a cancer by diagnosis fills in a report form, and is required to submit it to the OD within three days. Notification has been obligatory since the 1960s. The form contains guidelines for proper fill-out. For instance, the diagnosis is to be spelled out in words. If the local oncologist/doctor is uncertain about the diagnosis, the patient is to be referred to a larger hospital or the OD. For patients who are treated locally by a doctor who is not an oncologist, a medical report is submitted when a patient is discharged. The medical-report form also includes a field for description of the treatment and course of the disease. Both ICD-codes and words are used in the description. When the OD receives a medical report with incoherence between the two, the OD systematically contacts the local hospital for clarification.

In the submitted report, it is noted whether the patient is a resident of another borough than where he/she was diagnosed/treated. When a resident of AO is diagnosed elsewhere in Russia, the diagnosing hospital forwards the report to the OD in AO, and vice versa when non-AO residents are diagnosed in AO. Thus, reports of cancer cases among students and conscripts are forwarded to the regions in Russia where they have permanent residence.

When a person dies, a doctor submits a medical document to the local administrative office, which issues a death certificate and a certificate about identity and citizenship (no burial can take place before a death certificate is issued). The certificates are forwarded to the AO statistical office. The cause of death is established either on the basis of the deceased's anamnesis, or by pathological or forensic examination. Only one cause of death is recorded on the certificate, and the statistical office defines the ICD-code. Should the recorded cause of death be changed later, a notification is submitted to both the administrative and statistical offices. The family of the deceased can object to, or demand, a post-mortem examination. If a pathological examination reveals cancer, then both the stage and metastasis are made note of on the certificate. Medical employees from the OD visit the statistical office monthly to pick up information about cancer deaths. The OD then requests information about the revealed individuals from the registrar's office. This information is checked against the AKR to find out whether the specific case already has been registered. The information from the registrar also reveals whether an individual has changed surname between the time of diagnosis and time of death.

The OD sends an annual report to the ministry in Moscow about cancer incidence and mortality in AO. The report also includes the number of cases that was verified histologically and clinically. The OD is required to verify unverified cases within two years.

### Cancer registration

In 1996, a computer programme was put into use in the clinical work due to a directive from the health ministry. Data about cancer cases had until then only existed on paper-based patient records that were organised in an archive. The work to set up the AKR started in 1998. A data-entry programme was made using Access^®^, and all reported cases of cancer since 1993 were registered retrospectively. From 2001, the cancer cases were registered as soon as reports were received. The computer programme for the registration has been revised and improved twice since the registration started. Simultaneously, the information registered and the number of database fields included was modified (see Table 1). The electronic cancer registry has been administered and run by the OD.

The registration in the AKR was based on the information obtained on submitted form(s), and was updated if revised information was reported later. The oncologist in charge of the AKR routinely checked that reported histology and diagnosis were in accordance, and set the ICD-code if it was missing. The diagnosis was registered both in words and with a three character ICD-code. ICD-9 codes were used in registration of cases from the period 1993 – 99, and ICD-10 from year 2000. Thus, in the registered ICD-9 codes it was not possible to separate cancers in the sigmoid junction and rectum, so in the present study sigmoid junction was included in the rate estimate of rectum cancer – and not of colon cancer.

The data registered about each case are presented in Table 1. Not all of the data items were considered a must. But the following information has been obligatory (and inquired for if missing on the submitted form): name, patronymic, surname, gender, date of birth, place of residence, diagnosis, date of diagnosis, histology, stage according to the TNM-classification, method of diagnosis and treatment, place of treatment, and whether the patient is alive or dead. If dead, the following information was required: date of death, cause of death, and whether a pathological examination had been conducted. The identification of an individual was based on name, surname, patronymic and birth date.

The AKR registered whether a tumour was first revealed through autopsy, or if a case was first reported through a death certificate. The outcome of the cancer disease was also registered. The patients' last known status was updated annually. The updating was based on a final-treatment report, or on the patient's registration card if treated at the OD. One of the following eight possible outcomes was noted: alive; dead due to the treatment, the cancer, or another cause; treated basal cell carcinoma (and 5 years have passed without a relapse); moved to another region of Russia or abroad; the diagnosis showed to not be cancer; or unknown. The method of diagnosis was registered as one of the following: only clinical; histological; cytological; isotopic; endoscopic; x-ray; electronic tomography; ultra sound; surgery; or unknown. There were tick-off boxes on the report form for six of the methods, while the others usually were noted if used.

The registration procedures in the AKR included some internal quality control systems. After entry; the name, age, borough and diagnosis were checked visually against the received information to ensure correctness. The database programme used since year 2000 automatically recognises whether an individual has been registered before if the following data are identical: name; patronymic; surname; gender and birth date. In addition, the following logical checks are embedded in the programme: 1) that the birth date precedes the date of diagnosis in terms of real time; 2) that the date of diagnosis precedes the date of treatment/surgery; 3) that gender-specific diagnoses are logical in terms of registered gender. The latter was previously done by visual checks. Visual checks were also done to look for contradictions between diagnosis and histological verification and stage, respectively. Furthermore, most data-entry fields are formatted, which ensures that impossible or undefined values cannot be entered. In fields where a numerical value represents some defined information, the field is linked to a drop-down window that depicts and explains the range of possible values that can be entered. The fields that are formatted are depicted in Table 1.

### Quality and content assessment

To assess the completeness and correctness of the AKR, two of the authors (JAL and AV) visited two district hospitals (in Severodvinsk and Kholmogory) to check whether the living cancer patients in their files had been registered. The two hospitals were given a one-day notice before the control. The researchers were given full access to the cancer-patient files. In Severodvinsk 42 out of 723 and in Kholmogory 61 of 396 journals of living cancer patients were randomly selected and looked for in the AKR database. In addition, in Severodvinsk the following specific items were checked against the registry for each of the selected journals: name; year of birth; diagnosis; and date of diagnosis. These data were read aloud from the hospital files by JAL and checked in the database records by AV. The assessment revealed that six of the cancer patients residing in Severodvinsk were not registered. One of the 42 could not be checked because the person was not a resident of the borough. Out of the 59 who were residents of Kholmogory, none were missing in the AKR. Thus, overall 6 out of 100 (6 %) cancer cases belonging to the population were not registered (95% CI: 1.3–10.7%).

The data obtained in Severodvinsk were also used to assess the data-entry error in the AKR. In two of the checked files (5.7%), the diagnosis registered differed from the diagnosis in the journal. Both were recorded as ICD-10 C19 in the files, but registered as C20 and C50 in the AKR. One surname was incorrectly registered (1%), while for three cases there was a discrepancy in year of birth (3%). The registered year of diagnosis was in accordance with the data in all the checked journals. One record was missing the year of diagnosis and one other was missing the birth year, but the data were recorded in the patients' files.

The data-entry error rate was also assessed by checking the consistency between registered gender and gender-specific diagnoses (ICD-9: 174, 175, 180, 182, 183, 185 and 186) (this check was embedded in the computer programme from year 2000). There was a discrepancy between registered gender and diagnosis in one out of 4294 records (one case with the diagnosis ICD 174, female breast cancer, was registered as a male). The quality of the AKR was further assessed by looking at distributions of data; the proportions of missing values; the presence of non-logical values; and the proportion of registered cases whose case information was obtained from a death certificate.

In September 2001, Helge Stalsberg, a professor in morphology at the University of Tromsø and head doctor of the Department of Pathology at the University Hospital, was invited to Arkhangelsk to review the histological-diagnostic practise. Stalsberg was given full access to the material, including protocols concerning the diagnoses given for each case. He selected *a priori *to review the slides of the 20 most recent cases of each of the following cancer types: lung; stomach; colon; breast; and testes. Without consulting the protocol, he set a diagnosis one by one. His diagnoses were subsequently compared with the protocolled diagnoses. For testis cancer, only six cases were reviewed, as only six cases had been diagnosed so far that year. Thus, in total 86 cases were reviewed – all from 2001. The quality of the histological sections was considered adequate. There was full agreement concerning presence of invasive carcinoma in all cases of stomach and testis cancer. For both breast and colon cancer there were agreements for 19 of 20 slides. One of each was in disagreement on *in situ *versus invasive. For lung cancer, 2 of 20 were in disagreement on *in situ *dysplasia versus no dysplasia. The microtomes and microscopes possessed by the laboratory were found fully adequate for histological-diagnostic work.

## Results

A total of 35 224 cases of cancer, diagnosed in the period 1993 – 2001, were registered in the AKR. For 13 of the cases, the diagnosis had later been changed to non-cancer, while 42 cases were benign, in-situ carcinoma or secondary cancers. In addition, 472 registered cases had been revealed through autopsy. Thus, 34 697 cases were included in the calculations of cancer incidence. Of these, 95.2 percent had never had cancer before, and men constituted 51.3 percent. The number of new cases varied from 3559 in 1996 to 4153 in year 2000. In terms of age, the mode was 67 years, the median 63 years, and the range 0 – 99 years.

The annual proportion of diagnoses that were verified histologically was around 60 percent, while the proportion verified cytologically increased from 15 to around 20 percent in the studied period. The highest proportion verified histologically was for rectal cancer (81.4 %), while breast cancer was the cancer type most likely to be verified cytologically (36.8 %). (These figures include cases that were registered based on death certificates). The highest proportion verified by one of these two methods was in the age group 20 – 29, and the proportion was lower the older the age group.

**Figure 2:**
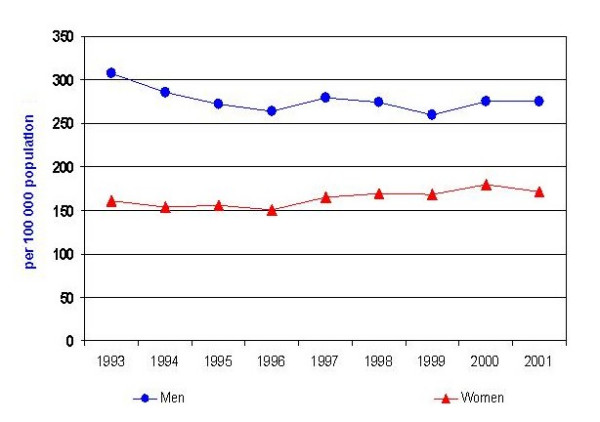
Age-adjusted, gender-specific incidence of cancer, all sites combined, in Arkhangelskaja Oblast.

**Figure 3:**
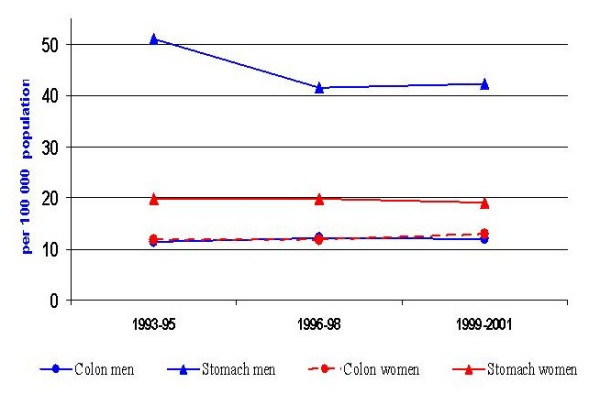
Trend of age-adjusted colon and stomach cancer among men and women in Arkhangelskaja Oblast.

**Figure 4:**
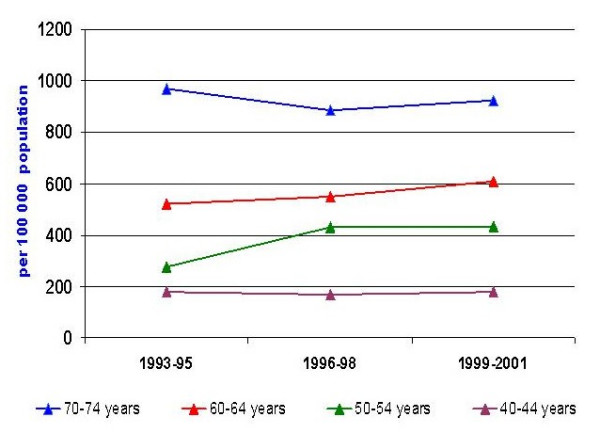
Trend in age-group specific cancer incidence among women in Arkhangelskaja Oblast, all sites combined.

**Figure 5:**
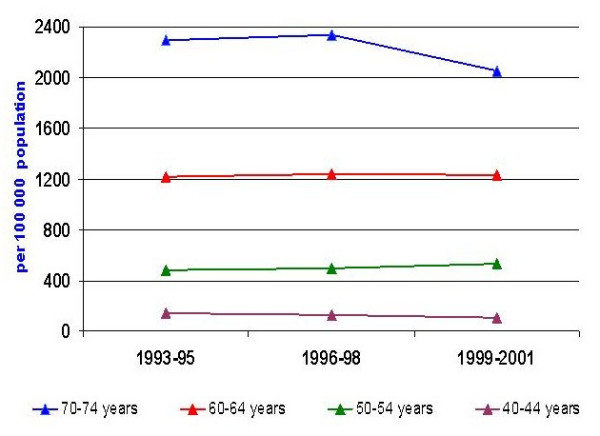
Trend in age-group specific cancer incidence among men in Arkhangelskaja Oblast, all sites combined.

The age-adjusted incidence rate for all sites combined was 164/100 000 for women and 281/100 000 for men. The highest incidence was cancer of the trachea, bronchus and lung (16.3% of all cases), whereof 88.6 % of the cases were among men. The second most frequent was stomach cancer (14.7%). Among women, cancer of the breast constituted 15.9 percent of all cases. The age-adjusted gender-and site-specific incidence rates are presented in Table 2. The cancer sites with the highest rates among men were: lung (77.4/100 000); stomach (45.9); rectum (13.4); oesophagus (13.0); and colon (12.2). Among women they were: breast (28.5); stomach (19.7); and colon (12.2). Some trends of cancer incidence are presented in Figures 2, 3, 4, 5, 6.

## Discussion

According to MacLennan (IARC), the following information is considered basic in a population-based cancer registry: date of diagnosis; basis of diagnosis; site of primary tumour; morphology; behaviour of tumour; source of information; place of residence; ethnic group; and personal information sufficient to ensure the recognition of an individual when the same person is reported more than once to the registry [8]. All these items were registered in the AKR, except for ethnicity. Ethnicity could be of interest to register in AO due to the Nenets indigenous population (0.5% of total population), but this was made unattainable when the Russian Federation implemented the use of "Russian" as nationality for all in identity documents. No unique identification number existed in Russia, but the identification of the individual was fulfilled in the AKR by registering the full name, gender and birth date.

### Quality

The AKR is a comprehensive registry in terms of important information about each cancer case, and had few data-entry errors. The pathology department at the OD works according to the classifications of the WHO International Histological Classification of Tumours, and the diagnostic procedures, equipment and skills were found to be fully adequate.

The correctness of incidence rates is influenced by the completeness of cover. The numerator in the rate estimates is deflated if not all cases of cancer in the official resident population were captured by the health system, and/or if the AKR did not receive reports about all diagnosed cases. The denominator, on the other hand, is incorrect if the health system of AO covered more or fewer people than the official population figures. The results of the controls carried out in Severodvinsk and Kholmogory indicate that the submission of cancer reports to the AKR was not fully complete, and that the reporting varied from district to district. There was no apparent explanation for the difference in reporting between the two clinics, which both had an oncologist. Incomplete reporting has also been found to hamper the estimations of cancer incidence in established systems with a long tradition of population based cancer registration, such as in Norway [9]. On the other hand, a fully complete cancer registry is inconceivable.

The AKR is a population-based cancer registry that is meant to cover the population of AO. Conscripts and students from elsewhere were not included in the official population figures, and cancer cases among them were not registered. There were two sub-groups of the official population of which some members were likely to survive, or die, with an undiagnosed cancer, and thereby contribute to an underestimation of incidence rates; namely, the indigenous Nenets and the poor elderly. The former mainly live in the north, and those among them who lived traditionally on the tundra as herders and hunters were less likely to seek or receive medical care from the public health care system. Poverty in Russia increased markedly in the 1990s – especially among elderly. Although health services in Russia were free and without service fees, the transport to the doctor or hospital usually had to be paid by the individual. Distances in rural areas are vast, and travelling was often uncomfortable and relatively expensive. Persons of age 80 years or older had the opportunity to transport without paying by inquiring for a requisition in advance. However, it is not unlikely that an increasing proportion of the elderly died from, or with, an undiagnosed cancer. Nevertheless, the age-specific incidence rates, all sites combined, in the older age groups did not decline within the study period.

Other sub-groups that were included in the official population figures, but possibly not fully reported to the AKR, were professional military personnel and railway and shipyard workers. These groups had their own health care privileges or systems that did not sort under the health authorities in AO, but directly under the state. This meant that patients who could not be treated within their own health system were sent to Moscow for treatment. Reporting was also done directly to Moscow, but this ended in the late 1990s for shipyard and railway workers. The OD had contact with the patients from these groups only if referred to them, and subsequently these patients would also be included in the AKR. The immediate families of these workers could also use the same health care system. The shipyard workers were located the city of Severodvinsk (population 230 000), which was a closed city due to the construction of nuclear and naval vessels at the shipyard. Figures about the number of workers in each of these groups were not publicly available, so the number of missing cases in the AKR from these two groups of workers is not readily estimated. But presumably the reduced allocation of funds after the disintegration of the USSR had the effect that fewer workers were sent to Moscow for cancer treatment. Furthermore, the retirement age for shipyard workers was as low as 45 years for women and 50 years for men, meaning that the workers were retired before they were in the highest risk groups in terms of age for most cancer types. The retirement age for male military personnel was 55 years.

Another group of concern in terms of population coverage was emigrants. People moving from AO to somewhere else in Russia were likely to register change of address, and if not, the report forms, if diagnosed elsewhere, would have been forwarded to AO. But quite a few people have emigrated to other former Soviet republics or other countries, and these people had no incentive or requirement to register. According to official figures there was a net emigration from AO of about 6000 people per year on average in the study period [10]. However, the first census since the disintegration of the USSR, which was held during the autumn of 2002, revealed that the actual population of AO was about 90 000 people lower than the official population figures, and that 2/3 of the deficit was among men [11]. Thus, the net emigration had been more than twice the official figures – on average. Based on age in 2002, the largest population discrepancies for men were in the age groups 35–39 and 60–64, and in the age groups 10–14 and 60–64 for women. The underestimation in the reported overall incidence rates for the whole study period should then be less than four percent due to this population factor. But since the population discrepancy varied with age group and gender, the underestimation will be of a higher magnitude for some cancer types and lower for others. The discrepancy between the official population-figures used in the present rate estimations and the real population size was presumably accumulative over time since the disintegration of the USSR. Hence, the influence of an inflated denominator on the estimated incidence rates was likely small in the early years of the study period, and more profound in the rates for 2000 and 2001.

The findings and notions discussed above indicate that the reported rates are underestimations, especially among men. However, the relative magnitudes of the site-specific cancer incidences within a gender would only have been affected by underreporting of cases if the population sub-groups discussed above were unusually prone, or less disposed, to certain types of cancer. Cancers related to the elderly were presumably of most concern in this perspective, but in the shipyard there might have been exposure to asbestos.

The proportion of cancer cases that was not captured by the health system, or diagnosed cases not caught by the AKR, can be estimated by calculating the annual proportion of cases among not-previously-registered individuals that were obtained from death certificates. The completeness of cover may also be evaluated by comparing the incidence rates of the different cancer types. The number of cases that were registered based on death certificate was 419 (2.4%) among men and 538 (3.0%) among women. The proportion varied from <0.6 percent in the years 1993–94 and 2000–01 to 5 percent in the period 1995–99. The actual proportion-level is an indication of how well the system works, and if the system worked consistently the proportion should have been fairly constant. Thus, the ability of diseased people to seek examination and treatment, and/or the function of the system, appears to have been impaired during the years of severe economic hardship. A study by Shkolnikov et al found that cancer deaths in the older age groups were under-recorded in Russia – especially in rural areas [12]. In Norway, the proportion of registered cases obtained from death certificates was 2.6 percent for both genders in 1993 [7].

The analysis revealed that almost 80 percent of all cases were verified histologically or cytologically. In the former USSR as a whole, 69.8 percent of the diagnoses were verified microscopically in 1989, and the proportion of cases verified varied from 62.4 to 81.3 percent between the different republics or oblasts within the union [1]. In the neighbouring country Norway, 86 percent of all cases since 1953 have been verified histologically [13].

### Cancer incidence

The age-adjusted cancer incidence among men, all-sites combined, was similar to the incidence in Norway. But among women the incidence was more than 30 percent lower in AO [13]. However, one should be careful in comparing the incidence rates in Russia with western countries since the competing risks of disease and death were different. The magnitude of the large difference in cancer incidence between men and women in AO was likely not due to an underestimation of rates, as the mentioned possible causes of underestimation mainly concerned men. For most cancers, the site-specific incidences found in AO were comparable both in magnitude and relative magnitude to rates for Russia in 1990, as reported by the International Agency for Research of Cancer (IARC) [14]. The total age-adjusted incidence among men was 284/100 000 in Russia vs. 267 in AO, while 170 vs. 151/100 000 among women (ICD-9: 173 was not included in these calculations). In comparison with the IARC-reported incidence, the site-specific incidence in AO among men was higher for oesophagus, and lower for larynx and testis cancer. Also women had a higher incidence of oesophagus cancer in AO, but the incidences of cancer of the lung, breast and cervix uteri were lower [14]. Compared to the incidence of female genital cancers in St. Petersburg, the incidences of corpus uteri and ovary cancer were lower in AO, while the incidence of cervix uteri cancer was of similar magnitude [15]. Interestingly, the rate of stomach cancer was relatively high and colon cancer low, just as in Norway 30–40 years ago when the rates were quite different from today [16].

The age distribution in AO, as in Russia, is different than the world standard population. Compared to the world standard (and most Western-European populations), there were relatively few older men and children below 10 years of age, as well as a small World-War II generation (age 50 – 59 in the study period). On the other hand, the post-war generation was relatively large (age 35 – 49). Thus, the distribution in AO was pear-shaped, in contrast to the pyramid-shape of the world standard population and the fairly evenly distribution of most Western-European populations. This means that the age adjustment of the incidence rates in AO contributed to an over-estimation of the cancer types that were most likely to develop in the age groups that were relatively small, and vice versa, compared to the actual burden of those cancers in the society.

The reported incidence rates in AO contribute to the discussion and generation of hypotheses concerning the role of different life-style and environmental factors in the aetiology of different cancer types. The AKR provides data for estimations and interesting insight to the cancer incidence in a northern Russian population. Using the registry for further investigations into district and age-group-specific differences in cancer incidence would shed additional light on the concerns and questions surrounding environmental and life-style aspects and cancer.

## Conclusion

We believe that the reported incidence rates reflect the cancer situation in AO well. Our findings then confirm and strengthen the indication that the incidence of melanoma of the skin and cancers of the stomach, larynx, liver, pancreas, prostate, colon, and bladder are quite different in male populations in Russia than, for instance, in Norway. Among women, most major cancer types, except stomach, appear to be relatively low in Russian populations.

## Competing interests

The author(s) declare that they have no competing interests.

## Authors' contributions

AV did the data analysis, quality assessment of the data and was the senior author. JAL was responsible for collecting the data, the data entry and the information about the health system and sub-populations. DSK designed the data entry programmes and extracted the database used in this study. AVT initiated the AKR, supervised the research group, and gave approval for the final version to be published. Sadly, AVT passed away not long thereafter. TSP made the AKR possible administratively. EL was the principal investigator, revised the article critically and gave approval of the final version.

## Pre-publication history

The pre-publication history for this paper can be accessed here:



## Figures and Tables

**Table 1:** Data items registered in the Arkhangelsk Cancer Registry

**Data registered**	**Comments**	
Number at registration card		
Surname, given name, patronymic	In separate fields from 1995	
Gender		C
Residence (urban or rural)		C
Date of birth	For the period 1995–99 the year was registered	
Rajon (borough) of residence		C
Home address	From 2000 also home oblast included	
Occupation		C
Date when registered as sick	(by general health system)	
Result of treatment / patient's status	Last known, at the end of the year.	C
Date of last status		
Clinical health group at the time of registration	e.g. *Will be cured, etc*. From 1995	C
Clinical health group at the end of year	From 1995	C
Descriptive diagnosis		
Cancer diagnosis ^1^	Only for the years 93–94	C
Diagnosis by ICD-9 code ^1^	Only for the years 1993–99	C
Diagnosis by ICD-10 code ^1^	Only for the years 1993–94, 2000-	C
How the tumour was detected	Registered if method was autopsy or only obtained from death certificate	C
Stage		C
TNM	From 1995	
Method of verification of diagnosis	Morphologic, cytologic, X-rays, endoscopy, isotopic, ultra sound, computer tomography, operation, only clinical, or unknown	C
Date of diagnosis	From 1995	
Reason for late diagnosis	From 2000	C
Morphological code		C
Main cancer site (when more than one site)	For internal use clinically	
Histological structure of tumour	Classification for internal use. From 1995	C
Synchronic tumour	Yes/no. Only in period 1995–99	
Localisation of cancer (side of body)	Right/left	C
Information about treatment	For the period 1995–s99	
Method of treatment, if radical	From 2000	C
Institution where diagnose was determined	From 2000	C
Institution where patient is under observation	Medical institution observing the patient	C
Metastasis?	Yes/no. Only in period 1995–99	
Number of tumours	From 2000	C
Date of last contact with patient	From 2000	
Cause of death	From 1995. Including code of cause from 2000	C
Date of death	From 1995	
Pathological examination	Yes/no. From 2000	C
Nationality	From 1995	C
Date cancer report received	From year 2000	

C = The field in the AKR database was formatted. I.e. data entry was restricted to possible or certain values.

^1 ^Three fields in the database, but in the period 1993–94 the values appeared in all three fields when data were entered in one of them.

**Table 2:** Cancer in Arkhangelskaja Oblast 1993–2001. Number of new cases and age-adjusted incidence rates per 100 000, by primary site and gender

			**Males**		**Females**	
						
	**ICD-9**	**ICD-10**	**Number new cases**	**Incidence rate**	**Number new cases**	**Incidence rate**
Lip	140	C00	266	4.2	95	0.7
Tongue	141	C01-02	109	1.6	27	0.2
Gum	143	C03	35	0.5	20	0.2
Floor of mouth	144	C04	88	1.3	5	0.1
Palate	145	C05-06	66	1.0	24	0.2
Salivary glands	142	C07-08	29	0.4	39	0.4
Tonsil / oropharynx	146	C09-10	168	2.4	42	0.4
Nasopharynx	147	C11	24	0.3	17	0.2
Periform sinus and other unspecified	149	C12, C14	58	0.8	4	0.0
Hypopharynx	148	C13	117	1.7	7	0.1
Oesophagus	150	C15	814	13.0	414	3.3
Stomach	151	C16	2880	45.9	2232	19.7
Small intestine	152	C17	32	0.5	27	0.3
Colon	153	C18	753	12.2	1346	12.2
Rectosigmoid junction, rectum and anus	154	C19-21	805	13.4	858	7.8
Liver	155	C22	325	5.1	221	2.0
Gallbladder and other unspecified	156	C23-24	82	1.4	183	1.5
Pancreas	157	C25	590	9.2	566	4.9
Other digestive organs	159	C26	2	0.0	4	0.0
Nasal cavity and paranasal sinuses	160	C30-31	58	0.8	49	0.5
Larynx	161	C32	472	6.9	33	0.4
Trachea, bronchus and lung	162	C33-34	5011	77.4	646	5.8
Pleura, thymus, mediastinum and heart	163,164	C37-38,C45	53	0.8	43	0.4
Other respiratory	165	C39	1	0.0	0	0.0
Bone, connective tissue, Kaposi's sarcoma	170	C40-41,C46	120	1.9	78	0.8
Melanoma of skin	172	C43	169	2.5	291	3.0
Other skin	173	C44	856	14.4	1478	12.9
Nervous system	192	C47,C70,C72	19	0.3	21	0.3
Retroperitoneum and peritoneum	158	C48	86	1.4	117	1.1
Connective and other soft tissue	171	C49	102	1.5	115	1.2
Breast	174,175	C50	20	0.3	2689	28.5
Vulva, vagina. other female genital organ	184	C51-52, C57		0.0	161	1.4
Cervix uteri	180	C53		0.0	790	8.4
Corpus uteri	182	C54		0.0	779	8.1
Uterus. unspecified	179	C55		0.0	3	0.0
Ovary	183	C56		0.0	854	9.0
Placenta	181	C58		0.0	11	0.2
Penis and other male genital organs	187	C60, C63	31	0.5		0.0
Prostate	185	C61	611	11.1		0.0
Testis	186	C62	110	1.5		0.0
Urinary tract, except bladder	189	C64-66,C68	592	9.1	574	5.7
Bladder	188	C67	737	11.6	223	1.8
Eye	190	C69	33	0.5	46	0.6
Brain	191	C71	303	4.4	289	3.7
Thyroid gland	193	C73	98	1.4	457	5.1
Adrenal and other endocrine glands	194	C74-75	27	0.4	18	0.2
Other or unspecified site	195,199	C76,C80	257	4.0	188	1.8
Hodgkin's disease	201	C81	184	2.7	158	2.1
Non-hodgkin's lymphoma	200	C82-83,C85	198	3.3	157	1.7
Other lyphoid and histiocytic tissue	202	C84,C96	18	0.3	10	0.1
Multiple myeloma	203	C88-90	70	1.1	96	0.9
Lymphoid leukaemia	204	C91	213	3.5	187	2.0
Myeloid leukaemia	205	C92	94	1.4	138	1.5
Monocytic leukaemia	206	C93	5	0.1	8	0.1
Other specified leukaemias	207	C94	21	0.3	25	0.3
Unspecified leukaemias	208	C95	26	0.4	39	0.4
